# CD4^+^ T Cells of Prostate Cancer Patients Have Decreased Immune Responses to Antigens Derived From SARS-CoV-2 Spike Glycoprotein

**DOI:** 10.3389/fimmu.2021.629102

**Published:** 2021-05-03

**Authors:** Pavla Taborska, Zuzana Strizova, Dmitry Stakheev, Ludek Sojka, Jirina Bartunkova, Daniel Smrz

**Affiliations:** ^1^ Department of Immunology, Second Faculty of Medicine, Charles University and University Hospital Motol, Prague, Czechia; ^2^ Department of Technical Operations, SOTIO, a.s., Prague, Czechia

**Keywords:** prostate cancer, SARS-CoV-2, COVID-19, HCoV-229E, spike glycoprotein

## Abstract

The adaptive immune response to severe acute respiratory coronavirus 2 (SARS-CoV-2) is important for vaccine development and in the recovery from coronavirus disease 2019 (COVID-19). Men and cancer patients have been reported to be at higher risks of contracting the virus and developing the more severe forms of COVID-19. Prostate cancer (PCa) may be associated with both of these risks. We show that CD4^+^ T cells of SARS-CoV-2-unexposed patients with hormone-refractory (HR) metastatic PCa had decreased CD4^+^ T cell immune responses to antigens from SARS-CoV-2 spike glycoprotein but not from the spiked glycoprotein of the ‘common cold’-associated human coronavirus 229E (HCoV-229E) as compared with healthy male volunteers who responded comparably to both HCoV-229E- and SARS-CoV-2-derived antigens. Moreover, the HCoV-229E spike glycoprotein antigen-elicited CD4^+^ T cell immune responses cross-reacted with the SARS-CoV-2 spiked glycoprotein antigens. PCa patients may have impaired responses to the vaccination, and the cross-reactivity can mediate antibody-dependent enhancement (ADE) of COVID-19. These findings highlight the potential for increased vulnerability of PCa patients to COVID-19.

## Introduction

The coronavirus disease 2019 (COVID-19) pandemic has significantly affected the global human population. The impact of the disease on the human population varies in different countries. It is affected by multiple factors, including different national preventive measures and population demographic factors such as age and ethnicity (1). The current epidemiological data already show that the more severe forms of COVID-19 do not impact the majority of the human population (2). However, large groups of people are at high risk of developing severe or even fatal forms of the disease. These groups are often people with other comorbidities, one of which is cancer (3, 4). Another significant factor that contributes to infection with severe acute respiratory system coronavirus 2 (SARS-CoV-2) and severity of COVID-19 is gender; men have been found to be more likely than women to become infected with the virus and to develop severe forms of the disease (5). Both of these risk factors, gender, and cancer, are combined in prostate cancer (PCa) patients. PCa is the second leading cause of cancer in men worldwide (6). The chances of PCa patients becoming infected with SARS-CoV-2 and developing a severe form of the disease are higher due to enhanced expression of the angiotensin-converting enzyme 2 (ACE-2) and the expression of the transmembrane protease, serine 2 (TMPRSS2) (4, 7).

A key role in the pathogenesis of COVID-19 is played by the immune system (8). Dysregulated adaptive and innate immune responses after infection with SARS-CoV-2 are thought to affect the severity of and mortality due to COVID-19 (9). The adaptive immune response is important for protection against viruses (10). It is also the key protective mechanism that is engaged after prophylactic vaccination (11). A recent study showed that the adaptive immune system of individuals who had been exposed to ‘common cold’ coronaviruses but not to SARS-CoV-2 were partially responsive to SARS-CoV-2-derived antigens (12). These findings suggested a T cell-mediated cross-reactivity between circulating ‘common cold’ coronaviruses and SARS-CoV-2. This cross-reactivity could be responsible for either enhanced protection against COVID-19 (13, 14) or, in contrast, for antibody-dependent enhancement (ADE) of COVID-19 (15, 16).

In this study, we investigated the adaptive immune cell responses of CD4^+^ and CD8^+^ T cells from SARS-CoV-2-unexposed individuals to spike glycoprotein antigens from SARS-CoV-2 (17) and the ‘common cold’ human coronavirus 229E (HCoV-229E) (18). We analyzed samples from 25 subjects, 14 of whom were hormone-refractory (HR) metastatic PCa patients and 11 of whom were healthy male volunteers. The adaptive immune responses were examined *in vitro* through the peptide-mediated enrichment of antigen-specific T cells in the subjects’ lymphocytes and the subsequent evaluation of the TNFα and IFNγ inflammatory response after peptide stimulation. The response rates, peptide cross-reactivity, and impact of PCa on the obtained data were evaluated.

## Materials and Methods

### Patients and Specimens

The source material from PCa patients was obtained *via* the leukapheresis samples or peripheral blood from 14 HR metastatic PCa patients obtained more than two years before the pandemic outbreak; between March 2011 and April 2017. In the group of 14 HR metastatic PCa patients, the median age was 67.0 years (range 52–74 years), the median Gleason score was 7.0 (Gleason range 7–9), and the prostate-specific antigen (PSA) level was 48.3 ng/ml (concentration range 0.8–701.4 ng/ml). The source material was also obtained from healthy male volunteers; 2 were obtained with leukapheresis, 4 were obtained from buffy coats, and 5 from peripheral blood. The leukapheresis samples obtained between November 2016 and April 2017 were within clinical projects sponsored by SOTIO, a.s. Other leukapheresis samples were obtained within the previous study (19). All patients provided signed informed consent for the use of their blood-derived products for future research. The buffy coats were obtained in October 2018 from the Institute of Hematology and Blood Transfusion in Prague. The peripheral blood samples of healthy male volunteers were obtained between June 2020 and March 2021. The volunteers were tested negative for the presence of antibodies specific to SARS-CoV-2 spike glycoprotein and reported no previous history of COVID-19 and positive tests for SARS-CoV-2. In the group of healthy male volunteers, the median age was 61.0 years (age range 29–78 years). Each donor provided signed written informed consent for the use of their blood-derived products for future research.

### Enrichment and Expansion of Antigen-Specific T Cells

Peripheral blood mononuclear cells (PBMCs) from leukaphereses and buffy coats were isolated as previously described (20). The isolated PBMCs were then cryopreserved in liquid nitrogen. The cryopreserved cells were reconstituted, and a 14-day enrichment with antigen-specific T cells was performed as previously described (21). For the enrichment of the reconstituted cells with antigen-specific T cells, a 1 μg/ml concentration of the following pooled overlapping peptide mixes spanning the indicated antigen was used: SARS-CoV-2 (17) [PepMix™ SARS-CoV-2 (Spike Glycoprotein), cat.# PM-WCPV-S-1, JPT Peptide Technologies, Berlin, Germany], and human coronavirus 229E (18) [PepMix™ HCoV-229E (Spike Glycoprotein), cat.# PM-229E-S-1, JPT]. As a positive control, pooled peptide mixes from Epstein-Barr virus (HHV-4), human cytomegalovirus (HHV-5), and influenza A (22) were used [1 μg/ml, PepMix CEF Pool (extended), cat.# PM-CEF-E, JPT].

### Cell Stimulation, Intracellular Cytokine Staining, and Cytokine Release

**Figure 1:**
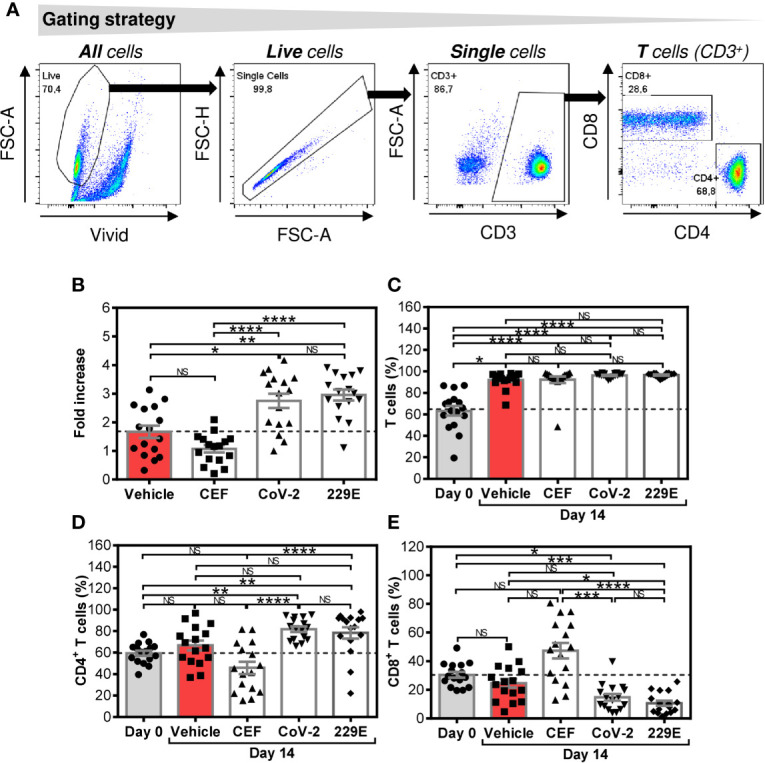
Changes in cell numbers and T cell population proportions after the SARS-CoV-2- and HCoV-229E-spike glycoprotein peptide pool-mediated culture enrichment **(A)** The gating strategy used to analyze flow cytometry data. **(B)** Cell number fold increase in cultures enriched with no peptides (Vehicle) or with CEF (CEF), SARS-CoV-2 (CoV-2), or HCoV-229E (229E) peptide pools. **(C)** Proportion of T cells (CD3^+^ cells) before (Day0) and after 14-day enrichment with the peptide pools described in **(B)**. **(D)** Proportion of CD4^+^ T cell population (CD3^+^CD4^+^ cells) before (Day0) and after 14-day enrichment with the peptide pools described in **(B)**. **(E)** Proportion of CD8^+^ T cell population (CD3^+^CD8^+^ cells) before (Day0) and after 14-day enrichment with the peptide pools described in **(B)**. In **(B–E)**, bars represent mean of values and SEM determined in each group and significances of differences among the groups are indicated (*P<0.05, **P<0.01, ***P<0.001, ****P<0.0001, *n* = 16 donors (6 healthy male donors and 10 HR metastatic PCa patients), 1-way ANOVA with the Dunn’s posttest). *P*>0.05 were considered nonsignificant (NS).

**Figure 2:**
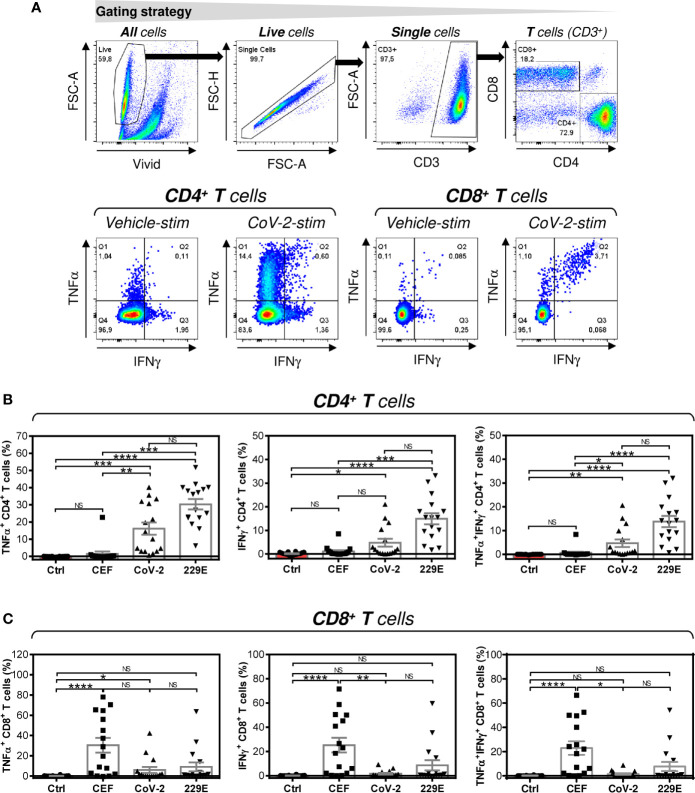
Peptide pool-mediated cell culture enrichment with the peptide-specific TNFα-, IFNγ-, or TNFα/IFNγ-producing CD4^+^ and CD8^+^ T cells **(A)** The gating strategy used to analyze flow cytometry data. The gating of CD4^+^ and CD8^+^ T cells (*top panels*). The gated CD4^+^ and CD8^+^ T cells stimulated without peptides (Vehicle-stim) or with SARS-CoV-2 (CoV-2-stim) peptide pool were gated to determine the proportions of TNFα- (Q1+Q2), IFNγ- (Q2+Q3), or TNFα/IFNγ- (Q2) producing cells (*bottom panels*). **(B)** Cell cultures enriched with CEF (CEF), SARS-CoV-2 (CoV-2), or HCoV-229E (229E) peptide pools were stimulated with the corresponding peptide pools and proportions of TNFα-, IFNγ-, or TNFα/IFNγ-producing CD4^+^ T cells was determined. As a control (Ctrl), the cell culture enriched with no peptide (Vehicle) and stimulated with the SARS-CoV-2 peptide pool was used. **(C)** Cells in **(B)** were analyzed for the proportions of TNFα-, IFNγ-, or TNFα/IFNγ-producing CD8^+^ T cells. In **(B, C)**, bars represent mean of values and SEM determined in each group and significances of differences among the groups are indicated (*P<0.05, **P<0.01, ***P<0.001, ****P<0.0001, *n* = 16 donors (6 healthy male donors and 10 HR metastatic PCa patients), 1-way ANOVA with the Dunn’s posttest). *P*>0.05 were considered nonsignificant (NS).

The cells were processed as described previously (21). Briefly, the cells were harvested, pelleted by centrifugation, and resuspended at a concentration of 1–4 × 10^6^ cells/ml in fresh human plasma serum-containing culture medium [LM medium; RPMI 1640 medium, 5% human plasma serum (One Lambda, Canoga Park, CA), 100 U/ml penicillin-streptomycin, 2 mM Glutamax, 1 mM sodium pyruvate and nonessential amino acid mix (Thermo Scientific)]. The cell suspension (200 μl) was transferred to a 96 U-bottom well plate (Nalgene, Rochester, NY). The cells were stimulated with 50 μl of LM media containing the pertinent peptides. The final concentration of the stimulating peptides in the cell suspension was 1 μg/ml. After 1.5 h of culture (37°C, 5% CO_2_), the cells were supplemented with brefeldin A (BioLegend, San Diego, CA) and then cultured for 4.5 h. Unstimulated controls (vehicle) were samples stimulated with the peptide solvent alone (20% DMSO in PBS). The cells were transferred to a V-bottom 96-well plate (Nalgene), stained with live/dead fixable stain, fixed, and permeabilized as previously described (23). The fixed and permeabilized cells were stained with the following antibodies: CD3-PerCP-Cy5.5, CD4-PE-Cy7 (eBiosciences, San Diego, CA), CD8-Alexa Fluor 700 (Exbio, Prague, Czech Republic), TNFα-APC, and IFNγ-PE (Becton Dickinson, Franklin Lakes, NJ) for 30–60 min at 4°C. The stained cells were washed with PBS/EDTA and analyzed by a FACSAria II (Becton Dickinson, Heidelberg, Germany). The obtained data were evaluated by FlowJo software (Tree Star, Ashland, OR). The frequency of responding T cells was determined by subtracting the frequency of the cytokine-producing T cells of the vehicle-stimulated sample from the frequency of the cytokine-producing T cells of the peptide pool-stimulated sample of the same patient or healthy volunteer. As a control (Ctrl), cell culture enriched with no peptide (vehicle) and stimulated with SARS-CoV-2 spike glycoprotein-derived peptides (peptide pools) was used. The gating strategy and determination of the cytokine-producing T cells are shown in **Figures 1A**, **2A**.

### Statistical Analysis

The means and SEM values were calculated from the indicated sample size (*n*) using GraphPad Prism 6 (GraphPad Software, La Jolla, CA). Statistical significance (*P<0.05, **P<0.01, ***P<0.001, ****P<0.0001) between two groups of differentially treated samples was determined by Wilcoxon matched-pair signed-rank tests and between three or more groups by matched-pair 1-way ANOVA with Dunn’s posttest. Statistical significance (*P<0.05, **P<0.01, ***P<0.001, ****P<0.0001) between two groups of subjects was determined by the Mann-Whitney *U* test. Correlations between two variables were assessed by Spearman’s rank-order correlation coefficient (r).

## Results

### SARS-CoV-2 and HCoV-229E Spike Glycoprotein-Derived Peptides Did Not Enrich the Cultured Cells With CD4^+^ or CD8^+^ T Cells

We first investigated how SARS-CoV-2 and HCoV-229E spike glycoprotein-derived peptides (peptide pools) affected the proportions of CD4^+^ and CD8^+^ T cells of healthy male volunteers and HR metastatic PCa patients during a 14-day culture. For this purpose, we used the culture protocol we previously used for culture enrichment with tumor-associated antigen-reactive T cells (21). As shown in **Figure 1B**, both the SARS-CoV-2- and HCoV-229E peptide pools promoted cell expansion compared with cells cultured without peptide pools (vehicle). The peptide pools had no effect on the proportion of the T cell (CD3^+^) population (**Figure 1C**). Both peptide pools also did not change the proportions of CD4^+^ and CD8^+^ T cells in the culture (**Figures 1D, E**). The positive control peptide pool CEF, which preferentially enriches cell cultures with Epstein-Barr virus-, human cytomegalovirus-, and influenza A-specific CD8^+^ T cells (22), did not have a significant effect on the cell count, T cell population enrichment, or changes in the proportions of CD4^+^ and CD8^+^ T cells compared with the unstimulated sample (vehicle) (**Figures 1B–E**). The results showed that both the SARS-CoV-2 and HCoV-229E-peptide pools comparably promoted enrichment of cell cultures with T cells without an impact on the CD4^+^: CD8^+^ T cell ratio.

### SARS-CoV-2 and HCoV-229E Spike Glycoprotein-Derived Peptides Comparably Enriched the Cultured Cells With Peptide-Specific CD4^+^ T Cells

Next, we investigated whether peptide-mediated enrichment with CD4^+^ T cells also led to enrichment with peptide-specific T cells. As shown in **Figure 2B**, the SARS-CoV-2 and HCoV-229E peptide pools comparably enriched the cell cultures with peptide-specific CD4^+^ T cells. As expected, the control CEF peptide pool had a negligible effect on the enrichment of the culture with peptide-specific CD4^+^ T cells. The CEF peptide pool substantially enriched the cultured cells with peptide-specific CD8^+^ T cells (**Figure 2C**). Fewer samples were enriched with peptide-specific CD8^+^ T cells by SARS-CoV-2 or HCoV-229E peptide pools (**Figure 2C**). These data showed that SARS-CoV-2 and HCoV-229E peptide pools predominantly enriched cell cultures with peptide-specific CD4^+^ T cells.

### HCoV-229E Spike Glycoprotein-Derived Peptides Enriched the Cultured Cells With CD4^+^ T Cells That Cross-React With SARS-CoV-2 Spike Glycoprotein-Derived Peptides

**Figure 3:**
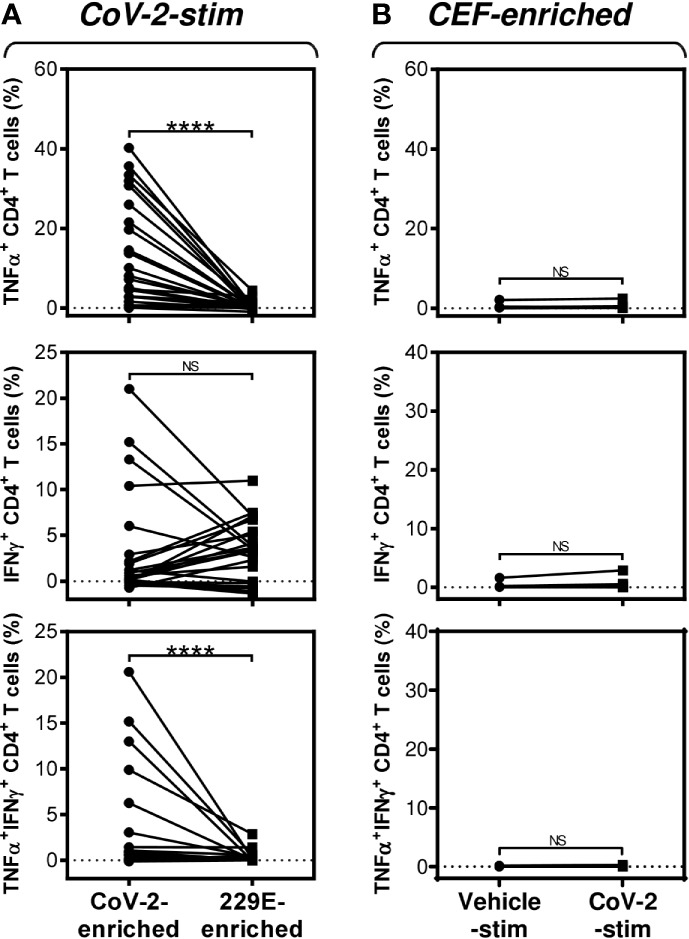
SARS-CoV-2/HCoV-229E reactivity and SARS-CoV-2 cross-reactivity of CD4^+^ T cells in cell cultures enriched with SARS-CoV-2- or HCoV-229E-spike glycoprotein peptide pools **(A)** The SARS-CoV-2 (CoV-2-enriched) or HCoV-229E (229E-enriched) peptide pool-enriched cell cultures were stimulated with SARS-CoV-2 peptide pool and differences in the proportions of TNFα-, IFNγ-, or TNFα/IFNγ-producing CD4^+^ T cells between both groups evaluated. **(B)** The CEF peptide pool-enriched cell cultures (CEF-enriched) were stimulated with no peptide (Vehicle-stim) or SARS-CoV-2 peptide pool (CoV-2-stim) and differences in the proportions of TNFα-, IFNγ-, or TNFα/IFNγ-producing CD4^+^ T cells between both groups evaluated. In **(A, B)**, significances of differences among the groups are indicated (****P<0.0001, A: *n* = 25 donors (11 healthy male donors and 14 HR metastatic PCa patients), B: *n* = 10 donors (2 healthy male donors and 8 HR metastatic PCa patients), Wilcoxon matched-pairs signed-ranks test). *P*>0.05 were considered nonsignificant (NS).

Previous studies have shown that T cells specific to the Dengue virus can mediate cross-protection against the Zika virus (13). Considering this mechanism, we investigated whether the cells that were enriched with the HCoV-229E spike glycoprotein-derived peptide pool also cross-reacted with the SARS-CoV-2 spike glycoprotein-derived peptide pool. As shown in **Figure 3A**, the HCoV-229E peptide pool enriched the culture with TNFα-, IFNγ- or TNFα/IFNγ-producing CD4^+^ T cells that cross-reacted with the SARS-CoV-2 peptide pool. The SARS-CoV-2 cross-reactivity in TNFα- or TNFα/IFNγ-producing CD4^+^ T cells was significantly lower than the SARS-CoV-2 reactivity of the SARS-CoV-2 peptide pool-enriched cells (**Figure 3A**, top and bottom panels). Surprisingly, the cross-reacting IFNγ-producing CD4^+^ T cells enriched with the HCoV-229E peptide pool had a comparable reactivity to the SARS-CoV-2 peptide pool as the IFNγ-producing CD4^+^ T cells enriched with the SARS-CoV-2 peptide pool (**Figure 3A**, middle panel). No SARS-CoV-2 peptide pool-reacting TNFα-, IFNγ- or TNFα/IFNγ-producing CD4^+^ T cells were found in the CEF peptide pool-enriched cell cultures (**Figure 3B**). These data showed that the HCoV-229E peptide pool could enrich cell cultures with SARS-CoV-2 cross-reacting CD4^+^ T cells.

### HR Metastatic PCa Patients Have Decreased CD4^+^ T Cell Responsiveness to SARS-CoV-2 but Not HCoV-229E Spike Glycoprotein-Derived Peptides Compared With Healthy Male Volunteers

**Figure 4:**
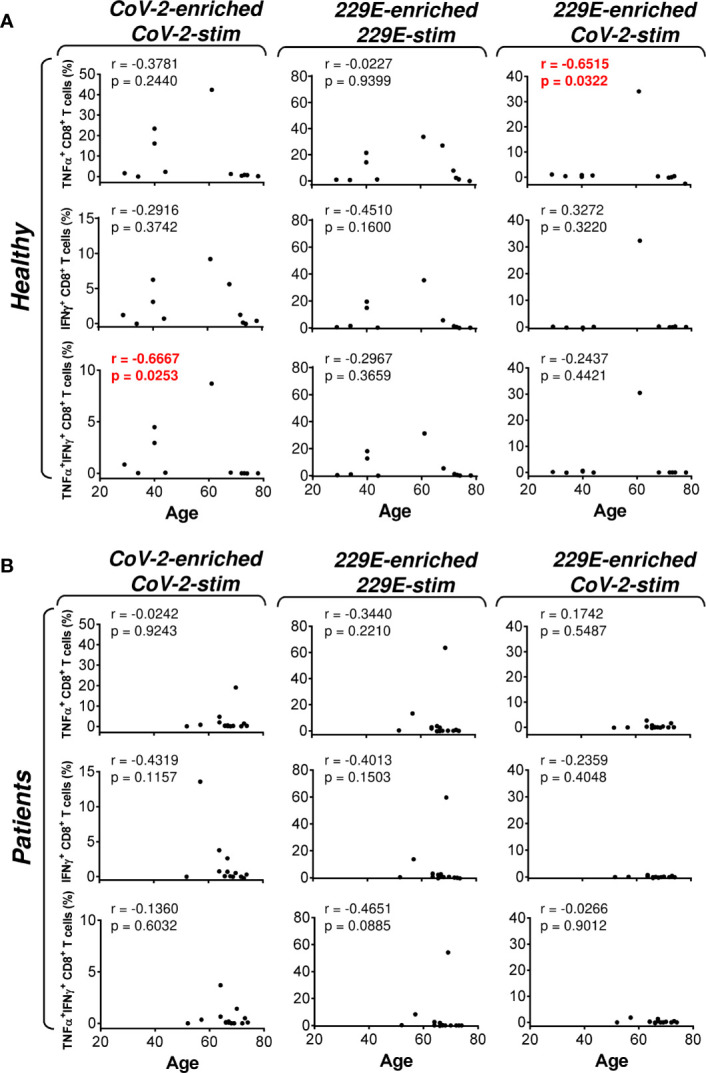
Association of the healthy male volunteer’s and HR metastatic PCa patient’s age with the SARS-CoV-2/HCoV-229E peptide pool reactivity or SARS-CoV-2 peptide pool cross-reactivity of CD8^+^ T cells in the peptide-enriched cell cultures **(A, B)** Cell cultures of 11 healthy male volunteers (Healthy) **(A)** and 14 HR metastatic PCa patients (Patients) **(B)** were SARS-CoV-2-enriched/SARS-CoV-2-stimulated (CoV-2-enriched/CoV-2-stim), HCoV-229E-enriched/HCoV-229E-stimulated (229E-enriched/229E-stim), or HCoV-229E-enriched/SARS-CoV-2-stimulated (229E-enriched/CoV-2-stim) and the correlations of the subject’s age with the proportions of TNFα-, IFNγ-, or TNFα/IFNγ-producing CD8^+^ T cells determined. In **(A, B)**, the correlations were evaluated by Spearman’s rank-order correlation coefficient (r) and the significance (*P* value) determined (*n* = 11 healthy male volunteers, *n* = 14 HR metastatic PCa patients). In **(A, B)**, *P* > 0.05 were considered nonsignificant.

**Figure 5:**
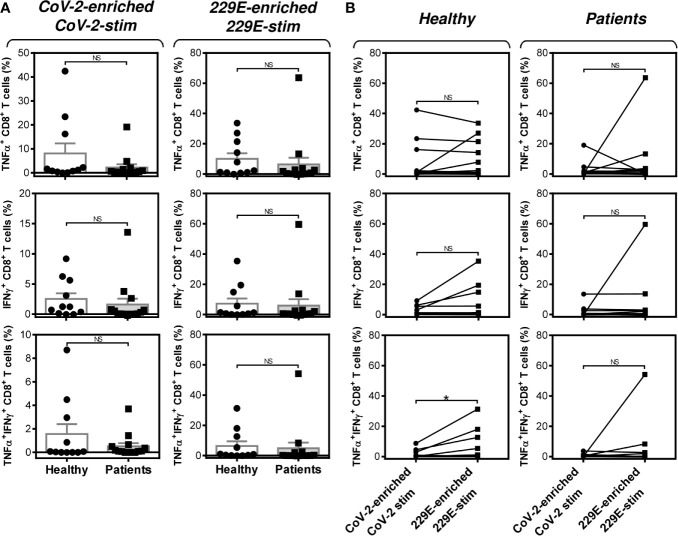
Impact of PCa on the peptide pool-mediated cell culture enrichment with the peptide-specific TNFα-, IFNγ-, or TNFα/IFNγ-producing CD8^+^ T cells **(A)** Cell cultures of 11 healthy male volunteers (Healthy) and 14 HR metastatic PCa patients (Patients) were enriched with the SARS-CoV-2 (CoV-2) (left) or HCoV-229E (229E) (right) peptide pool and stimulated with the corresponding peptide pool and proportions of TNFα-, IFNγ-, or TNFα/IFNγ-producing CD8^+^ T cells was determined. **(B)** The differences in responsiveness of CD8^+^ T cells to SARS-CoV-2 (CoV-2-enriched/CoV-2-stim) and HCoV-229E (229E-enriched/229E-stim) peptide pools in A was evaluated between the group of healthy male volunteers (Healthy) and HR metastatic PCa patients (Patients). In **A**, bars represent mean of values and SEM determined in each group and significances of differences between the groups are indicated (*n* = 11 healthy male donors (Healthy) and *n* = 14 HR metastatic PCa patients (Patients), Mann-Whitney *U* test). In **(B)**, significances of differences between the groups are indicated (*P<0.05, *n* = 11 healthy male donors (Healthy), *n* = 14 HR metastatic PCa patients (Patients), Wilcoxon matched-pairs signed-ranks test). In **(A, B)**, *P*>0.05 were considered nonsignificant (NS).

**Figure 6:**
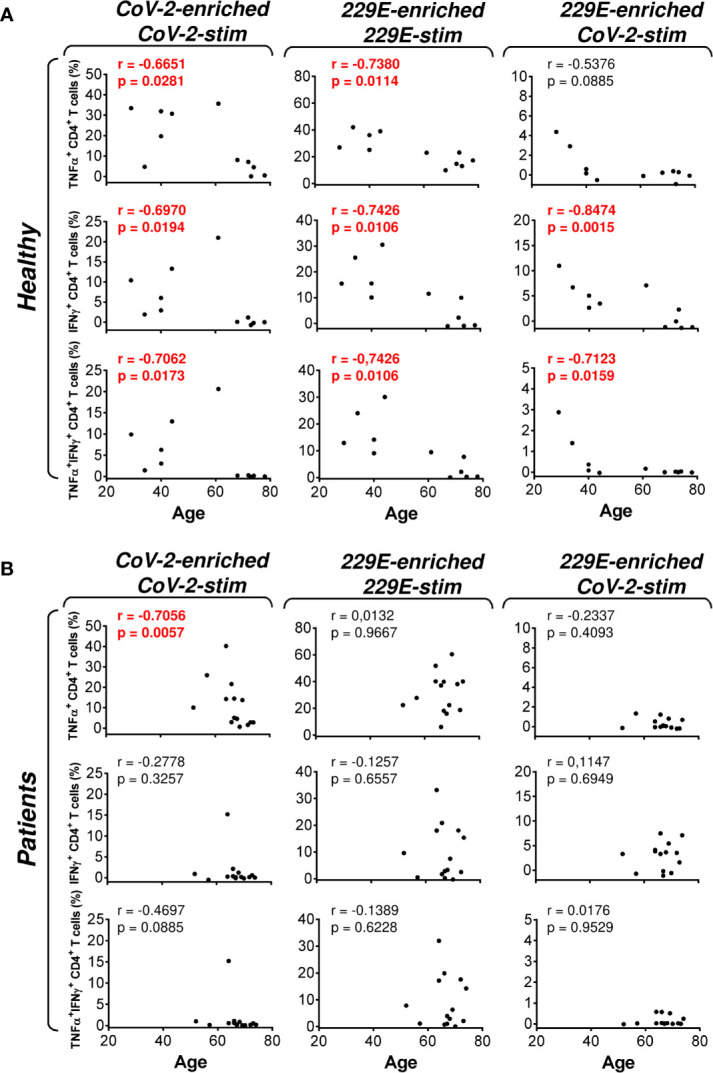
Association of the healthy male volunteer’s and HR metastatic PCa patient’s age with the SARS-CoV-2/HCoV-229E peptide pool reactivity or SARS-CoV-2 peptide pool cross-reactivity of CD4^+^ T cells in the peptide-enriched cell cultures **(A, B)** Cell cultures of 11 healthy male volunteers (Healthy) **(A)** and 14 HR metastatic PCa patients (Patients) **(B)** were SARS-CoV-2-enriched/SARS-CoV-2-stimulated (CoV-2-enriched/CoV-2-stim), HCoV-229E-enriched/HCoV-229E-stimulated (229E-enriched/229E-stim), or HCoV-229E-enriched/SARS-CoV-2-stimulated (229E-enriched/CoV-2-stim) and the correlations of the subject’s age with the proportions of TNFα-, IFNγ-, or TNFα/IFNγ-producing CD4^+^ T cells determined. In **(A, B)**, the correlations were evaluated by Spearman’s rank-order correlation coefficient (r) and the significance (*P* value) determined (*n* = 11 healthy male volunteers, *n* = 14 HR metastatic PCa patients). In **(A, B)**, *P* > 0.05 were considered nonsignificant.

As PCa can be associated with immunosuppression (24), we next examined whether HR metastatic PCa patients in our cohort had compromised responsiveness to SARS-CoV-2 and HCoV-229E peptide pools. We stratified our cohort into groups of healthy male volunteers and HR metastatic PCa patients and compared the results from our enrichment experiments between these two groups and the peptide pools. We found that the age of subjects in both groups had no large effect on cell culture enrichment with SARS-CoV-2- and HCoV-229E-reacting, or SARS-CoV-2-cross-reacting CD8^+^ T cells (**Figure 4**). We also did not find any differences in the responsiveness of CD8^+^ T cells between these two groups, nor the peptide pools (**Figure 5**). However, we found a significant difference in the responsiveness of CD4^+^ T cells to SARS-CoV-2 and HCoV-229E peptide pools between PCa patients and healthy donors. As shown in **Figure 6**, the age of healthy male volunteers had a large effect on cell culture enrichment with SARS-CoV-2- and HCoV-229E-reacting, or SARS-CoV-2-cross-reacting CD4^+^ T cells (**Figure 6A**). In the group of PCa patients, the patients’ age only affected cell culture enrichment with SARS-CoV-2-reacting TNFα-producing CD4^+^ T cells (**Figure 6B**), indicating that the impact of PCa patients’ age within the group of this study (52–74 years) did not largely contribute to the CD4^+^ T cell responsiveness to SARS-CoV-2- and HCoV-229E-derived antigens.

**Figure 7:**
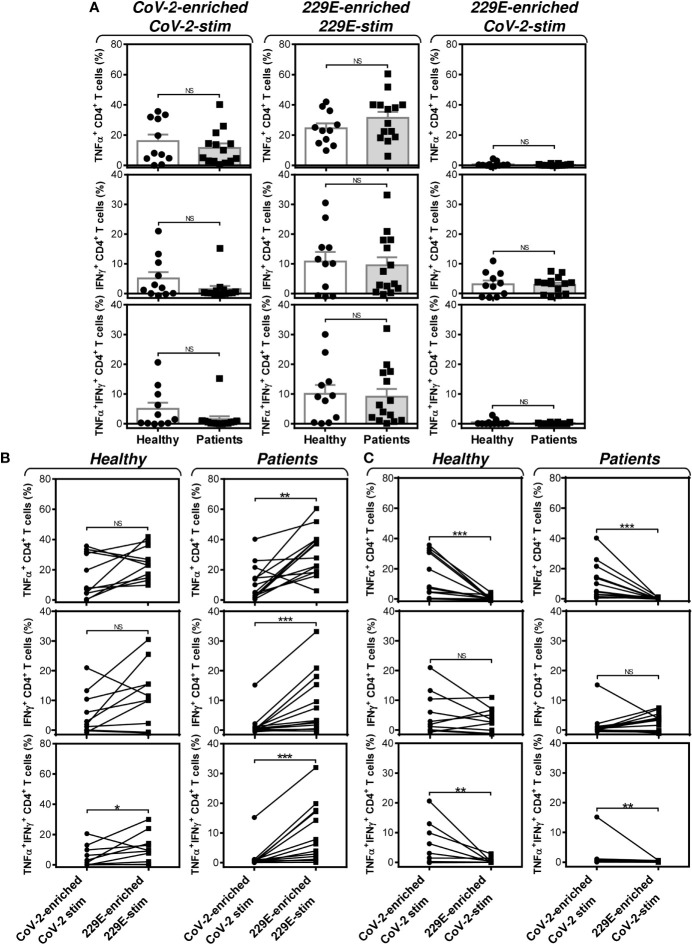
Impact of PCa on the peptide pool-mediated cell culture enrichment with the peptide-specific TNFα-, IFNγ-, or TNFα/IFNγ-producing CD4^+^ T cells. **(A)** Cell cultures of 11 healthy male volunteers (Healthy) and 14 HR metastatic PCa patients (Patients) were enriched with the SARS-CoV-2 (left) or HCoV-229E (229E) (middle and right) peptide pool and stimulated with the corresponding (CoV-2-enriched/CoV-2-stim, 229E-2-enriched/229E-stim) or non-corresponding (229E-2-enriched/CoV-2-stim) peptide pool and proportions of TNFα-, IFNγ-, or TNFα/IFNγ-producing CD4^+^ T cells was determined. **(B)** The differences in responsiveness of CD4^+^ T cells to SARS-CoV-2 (CoV-2-enriched/CoV-2-stim) and HCoV-229E (229E-enriched/229E-stim) peptide pools in **(A)** was evaluated between the group of healthy male volunteers (Healthy) and HR metastatic PCa patients (Patients). **(C)** The differences in responsiveness of CD4^+^ T cells to SARS-CoV-2-enriched/SARS-CoV-2-stimulated (CoV-2-enriched/CoV-2-stim) and HCoV-229E-enriched/SARS-CoV-2-stimulated (229E-enriched/CoV-2-stim) peptide pools in **(A)** was evaluated between the group of healthy male volunteers (Healthy) and HR metastatic PCa patients (Patients). In **(A)**, bars represent mean of values and SEM determined in each group and significances of differences between the groups are indicated (*P<0.05, **P<0.01, ***P<0.001, *n* = 11 healthy male donors (Healthy) and *n* = 14 HR metastatic PCa patients (Patients), Mann-Whitney *U* test). In **(B, C)**, significances of differences between the groups are indicated (**P<0.01, ***P<0.001, *n* = 11 healthy male donors (Healthy), *n* = 14 HR metastatic PCa patients (Patients), Wilcoxon matched-pairs signed-ranks test). In **(A–C)**, *P*>0.05 were considered nonsignificant (NS).

Further analyses revealed that the group of PCa patients responded similarly to the SARS-CoV-2 and HCoV-229E peptide pools as the group of healthy male volunteers (**Figure 7A**). However, the patients’ responsiveness to the SARS-CoV-2 peptide pool was compromised when compared to the HCoV-229E peptide pool’s responsiveness. As shown in **Figure 7B** (right panels), the patients’ responsiveness to the SARS-CoV-2 peptide pool was significantly lower than their responsiveness to the HCoV-229E peptide pool. On the other hand, TNFα- and IFNγ-producing CD4^+^ T cells of healthy male volunteers responded similarly to both SARS-CoV-2 and HCoV-229E peptide pools (**Figure 7B**, top left two panels). Only TNFα/IFNγ-producing CD4^+^ T cells responded significantly more to the HCoV-229E than the SARS-CoV-2 peptide pool (**Figure 7B**, bottom left panel). No difference between healthy male volunteers and PCa patients was found in the SARS-CoV-2 cross-reacting CD4^+^ T cell populations (**Figure 7C**).These data showed that the HCoV-229E peptide pool can partially rescue the patients’ compromised responsiveness to the SARS-CoV-2 peptide pool due to the HCoV-229E peptide pool-mediated enhanced enrichment of the patients’ cells with CD4^+^ T cells, which cross-react with the SARS-CoV-2 peptide pool.

## Discussion

In this study, we show how T cells of a SARS-CoV-2-unexposed population of HR metastatic PCa patients and healthy male volunteers responded to the pools of peptides derived from SARS-CoV-2 or HCoV-229E spike glycoproteins. The immune systems of the tested individuals were SARS-CoV-2-naive, and the levels of responsiveness of the CD4^+^ T cells to the SARS-CoV-2 and HCoV-229E peptide pools were comparable in the tested subjects. However, stratified analyses revealed that HR metastatic PCa patients had a significantly compromised responsiveness to the SARS-CoV-2 peptide pool compared to their responsiveness to the HCoV-229E peptide pool. This compromised responsiveness was partially rescued through the HCoV-229E peptide pool-mediated enrichment with CD4^+^ T cells that cross-reacted with the SARS-CoV-2 peptide pool.

During the current COVID-19 pandemic, there is an urgent need for the rapid development of treatments that could help contain the pandemic and prevent a much more devastating later waves of infection. One safety measure that can be used to control the pandemic is the identification of groups of people who are most vulnerable to the disease. The current epidemiological data show that men and cancer patients are relatively more vulnerable to the disease, as their chances of being infected and developing severe forms are much higher (3–5). PCa patients belong to both these groups (4, 7). In this study, we found that the immune systems of PCa patients may be less responsive to SARS-CoV-2- than to HCoV-229E-derived antigens, which can compromise the efficacy of anti-SARS-CoV-2 vaccines in these patients. This compromised responsiveness was not due to the inability of the patient’s immune system to mount an antigen-specific response in general because their immune cells were still able to develop a specific response to HCoV-229E- or CEF-derived antigens. These data, therefore, indicate that PCa patients might be able to generate a specific response to the viral diseases with which their immune system has presumably had previous experience, such as ‘common cold’ coronavirus (HCoV-229E), influenza, EBV, or HCMV. However, these patients may not efficiently generate responses to viruses to which their immune system has not been exposed, such as SARS-CoV-2.

The severity of COVID-19 has been shown to be associated with the age of the patients (25). Immunosenescence might play a role in the poor immunological response to SARS-CoV-2 antigens and contribute to the severity of COVID-19 (26). Our data indeed showed that CD4^+^ T cell response to both SARS-CoV-2-and HCoV-229E peptide pools negatively correlated with the tested individuals’ age, indicating that immunosenescence might contribute to the inefficient specific immune response of the elderly to coronavirus infections (27). Since PCa incidence is higher in elderly men, their age-related inefficient specific immune response to coronavirus infections and the compromised responsiveness to SARS-CoV-2 antigens can contribute to the vulnerability of these patients to SARS-CoV-2.

The SARS-CoV-2 spike glycoprotein is currently a focus of interest (28). Polyclonal antibodies specific to this protein were found to efficiently block the ACE-2-mediated entry of the virus into the target cells (29). Our data with peptides derived from this protein indicate that PCa patients might not have an adequate response to this protein when compared to the response of healthy male individuals. However, our data showed that peptides derived from HCoV-229E spike glycoprotein were not only able to elicit a response but also to stimulate the enrichment of cultured cells with IFNγ-producing CD4^+^ T cells that were cross-reactive with peptides derived from the SARS-CoV-2 spike glycoprotein. These data indicate that antigens from the HCoV-229E spike glycoprotein can elicit immune responses that, in the end, might lead to the production of antibodies that cross-react with the SARS-CoV-2 spike glycoprotein. This cross-reactivity does not necessarily need to mediate an enhanced protection against COVID-19 (30). In contrast, it may promote ADE of the disease (15), which is often associated with the infection of immune cells and leads to immune cell apoptosis (16). Severe forms of COVID-19 are associated with substantially decreased levels of immune cells (31). Whether other ‘common cold’ coronaviruses negatively contribute to the severity of COVID-19 through ADE towards the end of the ‘common cold’ season or whether exposure to these coronaviruses provides T cell-mediated protection that prevents the disease from developing into its severe or fatal forms remains to be elucidated.

In this study, we showed that PCa patients may represent a group of people who are potentially at high risk of developing severe COVID-19 due to their compromised ability to respond to SARS-CoV-2-derived antigens. In addition, their unaffected ability to respond well to ‘common cold’ coronaviruses and the findings that this response can cross-react with SARS-CoV-2 highlight the fact that ‘common cold’ coronaviruses play highly unpredictable roles in the COVID-19 pandemic, specifically in PCa patients.

## Data Availability Statement

The raw data supporting the conclusions of this article will be made available by the authors, without undue reservation.

## Ethics Statement

The studies involving human participants were reviewed and approved by The Ethics Committee of the University Hospital Motol in Prague. The patients/participants provided their written informed consent to participate in this study.

## Author Contributions

PT, ZS, DmS, and DaS conducted the experiments and/or analyzed the data. DaS designed the experiments. LS and JB supervised the sampling of human blood. DaS wrote the manuscript. PT, DmS, HS, ZS, LS, and JB contributed to the writing of the manuscript. DaS supervised the research. All authors contributed to the article and approved the submitted version.

## Funding

Research in the authors’ laboratories was supported by funding from Charles University – project GA UK No. 364218 and PRIMUS/MED/12, and funding from the Ministry of Health, Czech Republic – project AZV 16-28135A.

## Conflict of Interest

LS is a part-time employee of SOTIO, a.s., a biotech company developing cell-based immunotherapy. JB is a part-time employee and a minority shareholder of SOTIO, a.s.

The remaining authors declare that the research was conducted in the absence of any commercial or financial relationships that could be construed as a potential conflict of interest.
